# Dietary Bile Acids Improve Serum Antioxidant Status and Modulate Fecal Microbiota in Culled Ewes

**DOI:** 10.3390/ani16091367

**Published:** 2026-04-29

**Authors:** Dan Luo, Xinfeng Chen, Chang Liu, Kehui Ouyang, Mingren Qu, Qinghua Qiu

**Affiliations:** Jiangxi Province Key Laboratory of Animal Nutrition and Feed, College of Animal Science and Technology, Jiangxi Agricultural University, Nanchang 330045, China

**Keywords:** antioxidant capacity, bacterial microbiota, bile acids, fermentation characteristics, microbial diversity, ruminant production

## Abstract

This study aimed to investigate the effects of dietary bile acids (BA) supplementation on serum antioxidant capacity, fecal fermentation characteristics, microbial diversity, and community composition in culled ewes. Twenty 5-year-old culled Hu ewes with similar body weights (42.95 ± 1.07 kg) were randomly allocated to two groups (n = 10 per group). The control group (CON) received a basal diet, while the treatment group (BA400) was fed the same basal diet supplemented with 400 mg/kg BA. Compared with the CON group, the BA400 group showed enhanced serum activities of total antioxidant capacity, superoxide dismutase, and glutathione peroxidase, while also showing reduced concentrations of cortisol, malondialdehyde, and reactive oxygen species (*p* < 0.05). Fecal pH, ammonia nitrogen, total volatile fatty acids, and the concentrations and proportions of individual volatile fatty acids remained unaffected by BA supplementation (*p* > 0.05). Microbial analysis revealed that the BA400 group exhibited higher fecal bacterial richness and diversity than the CON group (*p* < 0.05). Analysis of similarities revealed significant differences between the CON and BA400 groups (R = 1.000, *p* = 0.007). Specifically, BA supplementation increased the relative abundances of beneficial taxa, including Verrucomicrobiota and *Akkermansia*, while decreasing potentially pathogenic bacteria such as Campylobacterota and Proteobacteria. These findings indicate that dietary BA supplementation improves serum antioxidant capacity and modulates fecal microbial diversity and community structure in culled ewes, suggesting that hindgut microbiota may contribute to the health benefits of BA supplementation in ruminant production.

## Simple Summary

Older breeding sheep often face health challenges from stress and free radicals, unstable molecules that harm body cells. This study investigated whether adding bile acids, natural compounds made by the liver to break down fats, could improve the well-being of these aging animals. Twenty 5-year-old female sheep received either a basal diet or one containing supplemented bile acids, and serum health indicators and fecal microbiota were examined. The supplemented sheep demonstrated stronger serum antioxidant capacity. Their feces also harbored more helpful bacteria and fewer harmful types, forming a healthier microbial balance. These results indicate that dietary bile acids supplementation can enhance antioxidant capacity and gut health in aging sheep. This work offers farmers an effective feeding approach to promote animal health and welfare in older flocks, supporting more ethical and sustainable ruminant production.

## 1. Introduction

Culled ewes leave the flock once their reproductive output falls below the economic threshold, after which carcass value declines predictably due to age-related physiological changes [1]. Repeated lactations lower basal metabolic rate and reduce the efficiency of nutrient use for lean-tissue accretion, while muscle becomes fine-fibered and perirenal fat expands [2,3]. Chronic muscle glycogen depletion produces dark, firm, dry meat at slaughter, compounding price penalties [3]. Fortunately, this decline can be arrested by a two-to-three-month finishing phase with energy-dense rations that re-establish positive energy balance and acceptable carcass conformation [4]. Therefore, short-term strategic supplementation of culled ewes is a low-risk, cost-effective route to recapture value that would otherwise be irretrievably lost.

Short-term finishing of culled ewes increasingly focuses on feed additives that correct the low-level digestive dysfunction typical of multiparous animals, rather than simply raising dietary energy or protein. Among these additives, bile acids (BA) have received attention because they facilitate lipid absorption and appear to moderate the mild, chronic inflammation that accompanies rapid fattening [5,6]. Supplementing a standard diet with a commercial BA product has been reported to improve milk yield, milk quality, and metabolic status in dairy cows and goats [7,8,9]. Dietary BA supplementation has also been shown to elevate intestinal lipase and lipoprotein lipase activities, attenuate hepatic lipid accumulation, improve serum antioxidant capacity, and enhance growth performance and carcass characteristics in poultry [5,6,10]. These reports indicate that dietary BA supplementation is a potential route to enhance production efficiency and ewe welfare without pharmaceutical intervention.

BA supplementation has been shown in monogastric models or human subjects to increase serum antioxidant capacity by concurrently remodeling the gut microbiota and activating host receptor pathways. Enrichment of beneficial taxa, such as *Lactobacillus*, *Bifidobacterium*, and butyrate-producing *Clostridia*, enhances microbial synthesis of antioxidant metabolites (notably butyrate, indole-3-lactate, and polyphenol derivatives) that enter the portal circulation, directly scavenge ROS, and upregulate Nrf2-dependent antioxidant enzymes [11]. In parallel, FXR and TGR5 signaling activated by BA downregulate NADPH oxidase while transcriptionally inducing superoxide dismutase, catalase, and glutathione peroxidase, thus further attenuating systemic oxidative stress [12,13]. However, our recent research demonstrated that BA supplementation ameliorates liver function without altering rumen microbial diversity or community in sheep [14], suggesting that BA may act through distinct mechanisms in ruminants—potentially via direct hepatic effects or hindgut microbial modulation rather than rumen manipulation. Whether these synergistic microbiota–host interactions occur in culled ewes undergoing metabolic and immune transition remains unknown. Rigorous studies that pair microbial profiling with comprehensive antioxidant biomarkers are therefore required to determine whether BA inclusion improves oxidative status via gut microbiota modulation in this economically important category of animals.

Accordingly, a 90-day feeding trial was conducted to determine whether BA supplementation could improve serum antioxidant capacity, alter fecal fermentation parameters, and reshape the fecal microbiota of culled ewes during intensive fattening. It was hypothesized that BA supplementation would improve serum antioxidant status, increase microbial richness, and maintain a more balanced community structure. The findings are expected to elucidate, from a fecal microbiota perspective, how BA supports the health of culled ewes and offers a novel framework for BA-assisted intensive finishing.

## 2. Materials and Methods

### 2.1. Experimental Design and Animal Feeding

All animal procedures were conducted in accordance with guidelines for animal welfare and were approved by the Animal Ethics Committee of Jiangxi Agricultural University (JXAULL-20240504). Twenty culled Hu ewes with uniform body weight (42.95 ± 1.07 kg) and age (60.55 ± 0.29 months) were randomly allocated to two groups (n = 10). Ewes in the control group (CON) received the basal diet only, whereas those in the treatment group (BA400) received the basal diet supplemented with 400 mg/kg BA. The ingredient composition and chemical analysis of the basal diet are shown in Table 1. The BA supplement was a mixture of hyodeoxycholic acid, chenodeoxycholic acid, and hyocholic acid (purity ≥ 98%). It was first diluted 1:20 (*w*/*w*) with ground corn; thereafter, it was thoroughly mixed with the remaining diet ingredients. The total trial lasted 90 d, with the first 20 d serving as an adaptation period to the basal diet, and the subsequent 70 d constituting the formal experimental period. The animals were housed in individual pens (3.0 m × 2.0 m) under natural thermoneutral conditions. Ewes were fed individually twice daily at 08:00 and 18:00, with clean drinking water available ad libitum. Daily feed allowance was adjusted to yield 5–10% orts based on the previous day’s intake.

### 2.2. Sample Collection and Analytical Method

Basal diet samples were collected on three randomly selected days each week, pooled, mixed, and ground for chemical analysis. During the last 7 days of the trial, feces were collected using the partial-collection method. Fresh feces were manually taken from the rectum of each ewe every 4 h to capture diurnal variations in hindgut microbiota while minimizing animal stress. This schedule yielded six spot samples per day and 42 per animal over the week. Each spot sample was immediately snap-frozen in liquid nitrogen and stored at −80 °C. Prior to analysis, equal aliquots from the 42 spot samples per ewe were pooled and lyophilized to create one representative fecal sample per animal. Two days before the experiment ended, blood was collected from the jugular vein into plain tubes before the morning feeding, and serum was obtained by centrifugation at 3000× *g* for 20 min. All samples were stored at −80 °C until analysis.

Crude protein, ether extract, calcium, and phosphorus in the basal diet were quantified according to AOAC [15] methods 2001.11, 945.16, 935.14, and 952.23, respectively. Neutral-detergent fiber (NDF) and acid-detergent fiber (ADF) were assayed as described by Van Soest et al. [16], with heat-stable *α*-amylase added throughout the procedure. Fecal fermentation characteristics serve as critical indicators of hindgut fermentation status and microbial metabolic activity. Following the procedure described by Sato and Nakajima [17], 5 g of feces was suspended in 20 mL of distilled water to prepare a fecal extract. The pH of the feces was measured immediately with a portable pH meter (testo 206, testo AG, Schwarzwald, Germany). The extract was centrifuged at 6000× *g* for 30 min, and the supernatant was collected. After a further centrifugation at 20,000× *g* for 30 min, the final supernatant was used for ammonia nitrogen (NH_3_-N) and volatile fatty acids (VFA) analyses. NH_3_-N was determined by the phenol-hypochlorite method. VFA were quantified by gas chromatography (Agilent 8860, Agilent Technologies, Inc., Santa Clara, CA, USA) against external standards, with instrument parameters and run conditions identical to those reported by Luo et al. [18]. Briefly, nitrogen was used as the carrier gas at a flow rate of 2.5 mL/min. The sample (1.0 μL) was injected in split mode (40:1) at 235 °C. The oven temperature program was as follows: 100 °C for 0.5 min, ramped to 180 °C at 10 °C/min and held for 2 min, and then ramped to 260 °C at 10 °C/min. Serum cortisol (COR) level was considered to be a stress indicator, and antioxidant indices monitored in this study included total antioxidant capacity (T-AOC), superoxide dismutase (SOD), glutathione peroxidase (GSH-Px), malondialdehyde (MDA), and reactive oxygen species (ROS). All parameters were quantified using assay kits from Beijing Sino-UK Institute of Biological Technology (Beijing, China), following the manufacturer’s instructions exactly. The oxidative stress index is calculated as the ratio of ROS to T-AOC.

### 2.3. DNA Extraction, Sequencing, and Analysis

Fecal microbial DNA was isolated with the QIAGEN PowerFecal Pro kit (QIAGEN, Hilden, Germany). Yield and purity were verified by Qubit 4 fluorometry (Life Technologies, Carlsbad, CA, USA) and NanoDrop ND-2000 spectrophotometry (Thermo Fisher Scientific, Waltham, MA, USA), respectively, and integrity was checked on 1% agarose gels. The bacterial 16S rRNA gene was amplified with primers 27F (5′-AGRGTTYGATYMTGGCTCAG-3′) and 1492R (5′-RGYTACCTTGTTACGACTT-3′), each carrying a unique 16 bp barcode. Reactions (25 μL) contained 5 μL of 5 × KAPA HiFi buffer, 1 μL of dNTPs (10 mM), 0.5 μL of KAPA HiFi HotStart polymerase (1 U/μL), 1 μL of each primer (10 μM), 2 μL of template DNA, and 14.5 μL of nuclease-free water. Thermal cycling began with 5 min at 95 °C, followed by 30 cycles of 95 °C for 30 s, 57 °C for 30 s, and 72 °C for 60 s, and ended with 5 min at 72 °C. Amplicons were purified with AMPure XP beads (Beckman Coulter, Brea, CA, USA) and quantified using the Quant-iT dsDNA HS Assay Kit (Invitrogen, Carlsbad, CA, USA). Libraries were constructed with the SMRTbell Express Template Prep Kit 2.0-SPv4 and sequenced on a PacBio Sequel II platform at BAXBio Technology (Beijing, China) after passing quality control. Owing to budget limitations and given that each ewe’s fecal sample was already a composite of 42 spot samples, the 10 individual fecal samples per group were randomly pooled in pairs to yield five composite samples per group, giving 10 composite samples in total for microbial analysis. Raw reads were deposited in the NCBI SRA under BioProject PRJNA1274616.

Raw PacBio HiFi circular consensus sequencing (CCS) reads were generated from demultiplexed data using SMRT Link (v11.0). Quality filtering was performed using DADA2 (v1.28) with the filterAndTrim function (parameters: maxEE = c(2,2), maxN = 0, minLen = 1300, maxLen = 1700). Denoising was subsequently performed using the dada function, followed by chimera removal using removeBimeraDenovo with the consensus method. Amplicon sequence variants (ASVs) were generated and served as the taxonomic units. Taxonomic assignment was performed by aligning ASVs to the SILVA 138 database with the classify-sklearn algorithm in QIIME 2. Alpha-diversity indices, including Richness, Chao1, ACE, Shannon index, Simpson index, and inverse Simpson index, were computed with the q2-diversity plugin. Between-group differences in community structure were examined by principal-coordinate analysis (PCoA) based on Bray–Curtis distances and statistically evaluated with analysis of similarities (ANOSIM) using the same dissimilarity matrix. Functional potential was inferred with PICRUSt2 (v2.6.0) to compare the predicted metagenomes of the CON and BA400 groups.

### 2.4. Statistical Analysis

Normality was evaluated with the Shapiro–Wilk test. Variables that were normally distributed or achieved normality after natural log-transformation (*p* > 0.05) were compared using Student’s *t*-test. The model is expressed as: $Y_{ij}=\mu_{i}+\epsilon_{ij}$, where $Y_{ij}$ is the observed value for the *j*-th individual in group *i* (CON or BA400), $\mu_{i}$ is the population mean of group *i*, and $\epsilon_{ij}∼N(0,\sigma^{2})$ is the random error. The null hypothesis $H_{0}:\mu_{CON}=\mu_{BA400}$ was tested against $H_{1}:\mu_{CON}\neq \mu_{BA400}$. Non-normal data were analyzed with the Mann–Whitney U test. All these procedures were performed in SPSS (v27; IBM, Chicago, IL, USA) with *α* = 0.05. Microbial biomarkers distinguishing the CON and BA400 groups were identified with LEfSe. Features differing significantly between the two groups were first detected by Kruskal–Wallis testing, verified by pairwise Wilcoxon tests, and then ranked by linear discriminant analysis (LDA) with an LDA score cutoff of 4 to reduce the false-positive rate.

## 3. Results

### 3.1. Serum Antioxidant Capacity

The effects of dietary BA supplementation on serum antioxidant capacity in culled ewes are presented in Table 2. Compared with the CON group, the BA400 group exhibited higher levels of T-AOC, SOD, and GSH-Px (*p* < 0.05). Conversely, the BA400 group showed lower levels of COR, MDA, ROS, and the oxidative stress index (*p* < 0.05).

### 3.2. Fecal Fermentation Characteristics

The effects of dietary BA supplementation on fecal fermentation characteristics in culled ewes are detailed in Table 3. BA supplementation did not affect fecal pH, NH_3_-N concentration, total VFA concentration, individual VFA concentrations, or their relative proportions (*p* > 0.05).

### 3.3. Fecal Alpha and Beta Diversity

**Figure 1:**
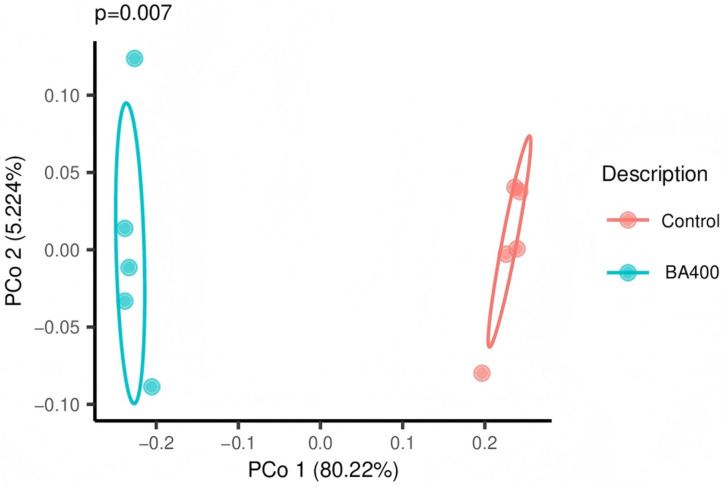
Effect of dietary bile acids supplementation on beta-diversity of the fecal bacteria based on principal coordinates analysis (PCoA). CON, the control group fed the basal diet; BA400, the treatment group fed the basal diet supplemented with 400 mg/kg bile acids.

Effects of dietary BA supplementation on fecal alpha-diversity metrics of culled ewes are listed in Table 4. The BA400 group exhibited higher Richness, Chao1, ACE, Shannon index, Simpson index, and inverse Simpson index than the CON group (*p* < 0.05). As shown in the PCoA plot (Figure 1), the CON and BA400 groups were clearly separated. ANOSIM revealed an R value of 1.000 and a *p*-value of 0.007 between the two groups.

### 3.4. Fecal Community Composition

Effects of dietary BA supplementation on fecal community composition at the phylum and genus levels in culled ewes are shown in Table 5 and Table 6, respectively. The BA400 group exhibited a higher relative abundance of Verrucomicrobiota than the CON group, whereas the relative abundances of Firmicutes, Campylobacterota, Cyanobacteria, Proteobacteria, Fibrobacterota, and Elusimicrobiota were lower (*p* < 0.05). At the genus level, *WCHB1-41*, *Akkermansia*, *Ruminiclostridium*, *p-2534-18B5 gut group*, and *F082* were more abundant in the BA400 group than in the CON group, whereas *Lachnospiraceae NK4A136 group*, *Bacteroides*, *Gastranaerophilales*, *UCG-002*, and *Succinivibrio* showed the opposite pattern (*p* < 0.05).

### 3.5. Fecal Marker Microbiota

**Figure 2:**
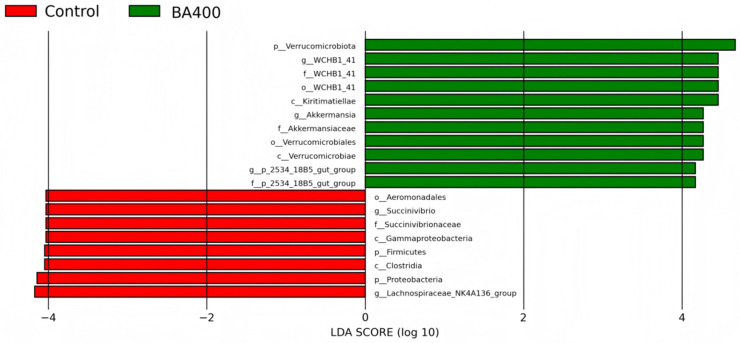
Effect of dietary bile acids supplementation on the microbial markers at different taxonomic levels based on linear discriminant analysis (LDA). CON, the control group fed the basal diet; BA400, the treatment group fed the basal diet supplemented with 400 mg/kg bile acids.

As shown in Figure 2, this study identified 19 marker microbes, with eight in the CON group and 11 in the BA400 group. The markers in the CON group were o__Aeromonadales, g__Succinivibrio, f__Succinivibrionaceae, c__Gammaproteobacteria, p__Firmicutes, p__Proteobacteria, c__Clostridia, and g__*Lachnospiraceae NK4A136 group*; the markers in the BA400 group were p__Verrucomicrobiota, p__WCHB1_41, c__WCHB1_41, o__WCHB1_41, c__Kiritimatiellae, g__*Akkermansia*, f__Akkermansiaceae, o__Verrucomicrobiales, c__Verrucomicrobiae, g__*p_2534_18B5 gut group*, and f__p_2534_18B5 gut group.

### 3.6. Fecal Metabolic Pathway

Effects of dietary BA supplementation on metabolic pathways predicted from fecal microbiota in culled ewes are presented in Table 7. In the BA400 group, protein families involved in genetic information processing, carbohydrate metabolism, translation, replication and repair, glycan biosynthesis and metabolism, biosynthesis of other secondary metabolites, sorting and degradation, and folding exhibited higher relative abundances than in the CON group (*p* < 0.05). Conversely, those associated with signaling and cellular processes and membrane transport were lower (*p* < 0.05).

## 4. Discussion

### 4.1. Effect of Dietary BA Supplementation on Serum Antioxidant Capacity

Culled ewes undergo physiological deterioration stemming from cyclic metabolic challenges associated with repeated gestation, which predisposes them to persistent oxidative stress and immunosuppression [3,4]. Consequently, bolstering antioxidant and immune status represents a pivotal strategy for optimizing their fattening efficiency. The current findings revealed that dietary incorporation of BA markedly ameliorated serum antioxidant capacity in culled ewes. These observations corroborate prior investigations documenting the beneficial effects of exogenous BA supplementation on antioxidant profiles and immunity in ruminants [7,19,20], even though the mechanistic underpinnings appear to involve multiple interacting pathways. Mechanistically, BA potentially triggers the farnesoid X receptor cascade, leading to transcriptional activation of antioxidant enzyme genes and subsequent augmentation of SOD and GSH-Px activities, thereby facilitating the scavenging of reactive oxygen species, including superoxide anions and hydrogen peroxide [13]. This mechanism parallels the work of Chen et al. [7], who demonstrated that 18 g/d BA fortification substantially elevated serum T-AOC, SOD, and GSH-Px concentrations in lactating dairy cattle. Moreover, the surfactant properties of BAs enhance micellar solubilization and intestinal uptake of fat-soluble vitamins [21], providing ancillary support to endogenous antioxidant mechanisms. Furthermore, BA-modulated hindgut microbiota enriches the pathway of biosynthesis of other secondary metabolites, enhancing conversion of complex polyphenols into bioactive metabolites. These polyphenol derivatives may activate the Nrf2-Keap1 pathway, upregulating endogenous antioxidant enzymes [11]. The observed reductions in circulating MDA and ROS concentrations, which are similar to the observations of Fan et al. [19] in transition dairy cows, signify effective quenching of lipid peroxidative damage and preservation of membrane structural integrity. This interpretation finds support in recent studies documenting BA-induced reductions in pro-inflammatory mediators in poultry models [22,23], underscoring the integrated anti-inflammatory and antioxidant functionality of these compounds in livestock species. The concomitant decrease in oxidative stress index substantiates that BA may operate through dual modalities, that is, fortifying antioxidant defenses while concomitantly curbing oxidant generation, to achieve holistic redox homeostasis.

### 4.2. Effects of Dietary BA Supplementation on Fecal Fermentation Characteristics

Current research regarding the effects of exogenous BA on fermentation characteristics has predominantly focused on the rumen, yielding inconsistent results. Yin et al. [24] and Li et al. [8] reported that BA supplementation did not affect rumen fermentation characteristics in lactating dairy goats and transition dairy cows, respectively. In contrast, Fan et al. [19], also investigating transition dairy cows, observed that dietary BA supplementation increased total VFA and acetate concentrations. These discrepancies likely stem from variations in the composition, proportion, and dosage of BA products employed across studies, given that individual BA components exert distinct physiological effects and that dose–response relationships differ substantially [7]. In goats with subacute ruminal acidosis, BA supplementation reduced total VFA concentrations while increasing isobutyrate levels in colonic digesta [20]. The present study, however, found that exogenous BA supplementation had no effect on fecal fermentation parameters. Collectively, these variations underscore the site-specific effects of BA on intestinal fermentation and highlight the necessity of considering BA composition and ratios when interpreting research findings and their practical applications.

### 4.3. Effects of Dietary BA Supplementation on Fecal Bacterial Diversity, Community, and Metabolic Pathway

The present study demonstrated that dietary supplementation with exogenous BAs altered fecal microbial diversity and community composition of culled ewes, consistent with previous findings in transition dairy cows [8,19]. Microbial community structure and function are intrinsically interdependent. Alpha diversity, encompassing richness and evenness components, can be rigorously assessed through richness indices (Richness, Chao1, and ACE) and diversity indices (Shannon index, Simpson index, and inverse Simpson index). While richness indices approximate the total species inventory, diversity indices capture taxonomic distribution balance. Collectively, these parameters illuminate community robustness and functional capability. Herein, dietary BA supplementation markedly elevated fecal microbial richness and diversity, findings concordant with Fan et al. [19]. The increased fecal microbial diversity following BA supplementation may be attributed to the interactive effects of BA and polyphenol derivatives on ruminal intestinal bacterial ecology. Polyphenols exhibit selective antimicrobial properties, suppressing pathogenic bacteria while promoting beneficial taxa [25]. BA-facilitated lipid emulsification enhances polyphenols’ release and absorption, amplifying their modulatory effects [26]. Undegraded polyphenols reaching the hindgut serve as prebiotic substrates, stimulating fermentation and cross-feeding interactions. This BA–polyphenol–microbiota interplay collectively contributes to the observed enhancement in hindgut bacterial diversity and community restructuring. Such enhanced diversity gains coincide with BA-induced gut health improvements and are accompanied by functional pathway enrichment, notably carbohydrate metabolism, glycan biosynthesis, and genetic information processing (translation, replication and repair, folding, and sorting and degradation). The upregulation of carbohydrate metabolism suggests enhanced microbial fermentation capacity and numerical increased VFA production, supporting improved energy harvesting during the fattening period. Concurrently, enriched glycan biosynthesis pathways imply stimulated mucin production, strengthening the intestinal mucus barrier and mitigating systemic oxidative stress. The activation of genetic information processing pathways indicates robust microbial proliferation and adaptive responses to BA-induced environmental shifts, collectively underpinning the previously observed growth performance [14] and current antioxidant benefits in culled ewes.

Species annotation provided more definitive evidence for fecal microbial alterations induced by dietary BA supplementation. Firmicutes constitutes one of the two most abundant bacterial phyla in the gut. Within this phylum, *Lachnospiraceae NK4A136 group* functions as a key butyrate producer, whereas F082 is primarily associated with propionate metabolism [27,28]. *Ruminiclostridium* contributes to the production of multiple VFA, including butyrate, acetate, and propionate [29]. Dietary supplementation with BA reduced the abundance of the butyrate-producing *Lachnospiraceae NK4A136 group*, while increasing *F082* and *Ruminiclostridium*, suggesting functional compensation among microbial communities to stabilize VFA production. *Akkermansia*, a beneficial gut bacterium of the phylum Verrucomicrobiota, plays pivotal roles in mucin degradation, intestinal barrier maintenance, short-chain fatty acid production, and metabolic health regulation, thus acting as a key hub connecting gut health with systemic metabolism [30]. Dietary BA supplementation elevated fecal abundances of Verrucomicrobiota and *Akkermansia*, indicating enhanced gut ecological function. Similarly, although *WCHB1-41*, *p-2534-18B5 gut group*, and *F082* are not yet fully characterized, their correlation with fiber fermentation, VFA production, enhanced barrier function, growth performance, and attenuated inflammation aligns with the broader evidence that fiber-adapted, VFA-producing gut bacteria confer benefits for intestinal and systemic health [31,32,33]. This further substantiated that dietary BA supplementation enhances intestinal micro-ecological health. In contrast, despite its capacity to ferment carbohydrates into acetic acid and succinic acid, *Bacteroides* has been implicated in various diseases, including metabolic disorders (e.g., obesity), immune-mediated conditions (e.g., inflammatory bowel disease), and colorectal cancer [34]. High-abundance taxa within the phyla Campylobacterota and Proteobacteria, including *Campylobacter jejuni*, Gastranaerophilales, *Salmonella*, *Vibrio*, *Pseudomonas*, and *Helicobacter*, are typically indicative of intestinal dysbiosis and inflammatory infections [35,36]. Notably, the aforementioned Campylobacterota and Proteobacteria exhibited elevated abundance in the CON group, suggesting that BA supplementation may suppress these potentially pathogenic microorganisms and enhance microbial community stability and host health. Interestingly, Fibrobacterota, which is specialized in cellulose degradation, displayed a similar trend, a finding contrary to the results of Luo et al. [18], who reported increased fecal abundance of this phylum following dietary resveratrol supplementation. Moreover, *Succinivibrio*, a microorganism capable of fermenting starch and soluble carbohydrates into succinate and acetate [37], also exhibited elevated abundance in the CON group. The underlying mechanisms may involve reduced fibrous material and starch availability in the hindgut due to enhanced fiber and starch degradation efficiency in the intestinal tract facilitated by BA supplementation [14]. These differentially abundant microorganisms suggest that BA supplementation may enhance intestinal microbial community diversity, stability, and functionality, specifically characterized by enrichment of beneficial taxa and concomitant suppression of potentially pathogenic bacteria.

It is important to note that the random pairwise pooling strategy used for microbial analysis, while reducing inter-individual variability and maintaining group-level representativeness, resulted in the loss of individual-level microbial resolution. Specifically, pooling precluded the assessment of within-group inter-individual variation and identification of responder and non-responder individuals to BA supplementation. Consequently, correlations between individual microbiota profiles and physiological parameters (e.g., serum antioxidant capacity and cortisol) could not be established, limiting our understanding of inter-individual heterogeneity in BA responses. Furthermore, pooled samples may mask outlier microbial profiles, indicating that observed group differences may not reflect responses in all individuals. Future studies should employ individual-based sequencing where financially feasible to capture comprehensive microbe–host interactions. The absence of metabolomic and transcriptomic data limits our ability to establish direct connections between microbial community shifts and host phenotypic responses. Future integration of metabolomics (targeting microbial metabolites and host-derived metabolites) and transcriptomics (focusing on intestinal and hepatic gene expression) is warranted to construct the BA–microbiota–metabolism–host health axis, elucidating the mechanistic underpinnings of BA-induced benefits in fattening culled ewes.

## 5. Conclusions

Dietary BA supplementation increased serum antioxidant capacity and fecal bacterial diversity, and altered the fecal microbial community. This study demonstrates that dietary BA supplementation improves the culled ewes’ health and modulates fecal microbial diversity and community composition, specifically characterized by enhanced fecal microbial diversity and enrichment of beneficial bacteria coupled with suppression of pathogenic bacteria.

## Figures and Tables

**Table 1:** Ingredient composition and chemical composition of the basal diet.

Ingredient	Proportion, g/kg	Chemical Composition	Value
Corn	480.0	Metabolizable energy, Mcal/kg	2.65
Soybean meal	90.0	Crude protein, %	13.02
Wheat bran	100.0	Neutral detergent fiber, %	26.55
Wheat straw	75.0	Acid detergent fiber, %	16.64
Peanut straw	225.0	Ether extract, %	3.19
Calcium hydrogen phosphate	7.50	Calcium, %	0.85
Limestone	7.50	Phosphorus, %	0.51
Salt	5.00		
Premix ^1^	10.0		

^1^ Premix provided the following per kg of DM: 1400 mg of Fe, 1200 mg of Zn, 250 mg of Cu, 900 mg of Mn, 100,000 IU of vitamin A, 27,000 IU of vitamin D3, and 800 IU of vitamin E.

**Table 2:** Effects of dietary bile acids supplementation on the serum antioxidant capacity of culled ewes.

Item	CON	BA400	SEM	*p*-Value
Cortisol (COR), ng/mL	42.81	31.49	0.793	<0.001
Total antioxidant capacity (T-AOC), U/mL	7.120	9.44	0.151	<0.001
Superoxide dismutase (SOD), U/mL	73.10	93.95	1.204	<0.001
Glutathione peroxidase (GSH-Px), U/mL	172.67	196.22	4.888	0.001
Malondialdehyde (MDA), nmol/mL	4.54	2.95	0.233	<0.001
Reactive oxygen species (ROS), U/mL	170.74	134.20	6.124	0.002
Oxidative stress index (OSI)	23.77	14.18	0.635	<0.001

Note: CON, the control group fed the basal diet; BA400, the treatment group fed the basal diet supplemented with 400 mg/kg bile acids; SEM, standard error of the mean.

**Table 3:** Effects of dietary bile acids supplementation on fecal fermentation characteristics of culled ewes.

Item	CON	BA400	SEM	*p*-Value
Fecal pH	7.53	7.45	0.037	0.157
Ammonia nitrogen, mg/dL	6.61	7.17	0.436	0.579
Total volatile fatty acids, mmol/L	29.88	30.96	1.591	0.641
Concentration, mmol/L				
Acetate	21.39	22.45	1.275	0.567
Propionate	5.91	5.88	0.169	0.904
Isobutyrate	0.34	0.33	0.017	0.708
Butyrate	1.74	1.81	0.133	0.738
Isovalerate	0.35	0.34	0.023	0.857
Valerate	0.16	0.16	0.020	0.975
Branched-chain volatile fatty acids	0.84	0.82	0.058	0.843
Proportion, %				
Acetate	71.30	72.38	0.550	0.190
Propionate	20.08	19.16	0.538	0.248
Acetate to propionate ratio	3.59	3.80	0.123	0.250
Isobutyrate	1.15	1.07	0.045	0.259
Butyrate	5.80	5.81	0.236	0.989
Isovalerate	1.16	1.09	0.052	0.457
Valerate	0.51	0.49	0.048	0.821
Branched-chain volatile fatty acids	2.82	2.66	0.117	0.380

Note: CON, the control group fed the basal diet; BA400, the treatment group fed the basal diet supplemented with 400 mg/kg bile acids; SEM, standard error of the mean.

**Table 4:** Effects of dietary bile acids supplementation on fecal alpha-diversity metrics of culled ewes.

Item	CON	BA400	SEM	*p*-Value
Richness	470.40	547.80	4.654	0.008
Chao1	504.35	569.48	4.959	<0.001
ACE	495.01	564.96	4.908	<0.001
Shannon index	5.47	5.76	0.016	0.008
Simpson index	0.9924	0.9949	0.0002	0.008
Inverse Simpson index	132.46	198.32	4.756	0.008

Note: CON, the control group fed the basal diet; BA400, the treatment group fed the basal diet supplemented with 400 mg/kg bile acids; SEM, standard error of the mean.

**Table 5:** Effects of dietary bile acids supplementation on fecal community composition at the phylum level in culled ewes.

Phylum Name	CON	BA400	SEM	*p*-Value
Bacteroidota	63.15	63.20	1.423	0.983
Firmicutes	19.68	17.27	0.675	0.038
Verrucomicrobiota	6.88	16.37	0.824	0.001
Campilobacterota	3.41	1.85	0.420	0.034
Cyanobacteria	2.36	0.66	0.084	0.008
Proteobacteria	2.72	0.04	0.301	0.008
Fibrobacterota	1.28	0.28	0.084	<0.001
Elusimicrobiota	0.36	0.13	0.026	<0.001
Spirochaetota	0.16	0.20	0.028	1.000

Note: CON, the control group fed the basal diet; BA400, the treatment group fed the basal diet supplemented with 400 mg/kg bile acids; SEM, standard error of the mean.

**Table 6:** Effects of dietary bile acids supplementation on fecal community composition at the genus level in culled ewes.

Genus Name	CON	BA400	SEM	*p*-Value
*Rikenellaceae RC9 gut group*	43.31	39.98	1.627	0.187
*Alistipes*	6.58	7.76	0.556	0.310
*WCHB1-41*	3.63	9.42	0.778	0.008
*UCG-010*	6.15	6.27	0.418	0.856
*Akkermansia*	3.25	6.96	0.431	0.003
*dgA-11 gut group*	4.55	5.57	0.313	0.055
*Prevotellaceae UCG-004*	3.49	3.42	0.249	0.849
*Lachnospiraceae NK4A136 group*	3.91	0.83	0.567	0.010
*Campylobacter*	2.88	1.85	0.375	0.111
*Bacteroides*	2.16	1.61	0.121	0.015
*Ruminiclostridium*	1.13	2.40	0.084	<0.001
*p-2534-18B5 gut group*	0.13	2.99	0.237	0.008
*Gastranaerophilales*	2.36	0.66	0.084	0.008
*UCG-002*	1.77	1.23	0.105	0.006
*F082*	1.11	1.76	0.144	0.017
*UCG-009*	1.40	1.33	0.127	0.693
*Succinivibrio*	2.13	0.00	0.309	0.008

Note: CON, the control group fed the basal diet; BA400, the treatment group fed the basal diet supplemented with 400 mg/kg bile acids; SEM, standard error of the mean.

**Table 7:** Effects of dietary bile acids supplementation on metabolic pathways predicted from fecal microbiota in culled ewes.

Metabolic Pathway	CON	BA400	SEM	*p*-Value
Protein families: genetic information processing	17.77	17.89	0.021	0.007
Protein families: signaling and cellular processes	9.95	9.62	0.074	0.024
Carbohydrate metabolism	8.81	8.88	0.011	0.003
Amino acid metabolism	7.42	7.50	0.039	0.189
Protein families: metabolism	6.41	6.43	0.007	0.184
Metabolism of cofactors and vitamins	4.36	4.40	0.014	0.095
Energy metabolism	4.28	4.33	0.018	0.056
Translation	3.60	3.67	0.010	0.001
Replication and repair	3.05	3.09	0.006	0.001
Nucleotide metabolism	2.62	2.63	0.004	0.690
Glycan biosynthesis and metabolism	2.25	2.31	0.007	0.001
Lipid metabolism	2.05	2.06	0.012	0.625
Signal transduction	1.89	1.87	0.008	0.095
Biosynthesis of other secondary metabolites	1.78	1.83	0.008	0.003
Membrane transport	1.72	1.59	0.027	0.020
Cellular community—prokaryotes	1.56	1.54	0.012	0.260
Sorting and degradation	1.53	1.54	0.004	0.011
Folding	1.53	1.54	0.004	0.011
Metabolism of other amino acids	1.24	1.24	0.006	0.989
Metabolism of terpenoids and polyketides	1.04	1.07	0.007	0.060

Note: CON, the control group fed the basal diet; BA400, the treatment group fed the basal diet supplemented with 400 mg/kg bile acids; SEM, standard error of the mean.

## Data Availability

Raw reads were deposited in the NCBI SRA under BioProject PRJNA1274616.

## Abbreviations

The following abbreviations are used in this manuscript:

BA	Bile acids
ROS	Reactive oxygen species
NDF	Neutral detergent fiber
ADF	Acid detergent fiber
NH_3_-N	Ammonia nitrogen
VFA	Volatile fatty acids
SOD	Superoxide dismutase
GSH-Px	Glutathione peroxidase
MDA	Malondialdehyde
ASVs	Amplicon sequence variants
PCoA	Principal-coordinate analysis
ANOSIM	Analysis of similarities
SRA	Sequence Read Archive
PICRUSt2	Phylogenetic investigation of communities by reconstruction of unobserved states 2
LEfSe	Linear discriminant analysis effect size
LDA	Linear discriminant analysis
